# Nomogram for predicting opioid-induced nausea and vomiting for cancer pain patients

**DOI:** 10.1007/s00520-023-08144-0

**Published:** 2023-11-02

**Authors:** Lingping Kong, Jing Wang, Shasha Guan, Xiaochen Chen, Meiqing Li, Liming Gao, Diansheng Zhong, Linlin Zhang

**Affiliations:** 1 Department of Medical Oncology, Tianjin Medical University General Hospital, Tianjin, 300052 China; 2 Tianjin Key Laboratory of Lung Cancer Metastasis and Tumor Microenvironment, Lung Cancer Institute, Tianjin Medical University General Hospital, Tianjin, 300052 China; 3 Department of Oncology, People’s Hospital of Inner Mongolia Autonomous Region, Hohhot, 010017 Inner Mongolia China; 4 Department of Oncology, The First Hospital of Qinhuangdao, Qinhuangdao, 066000 Hebei China

**Keywords:** Cancer, Opioid-induced nausea and vomiting (OINV), Nomogram, Predictive model, Risk factors

## Abstract

**Objective:**

Opioid-induced nausea and vomiting are frequently observed as an adverse effect in the treatment of cancer-related pain. The factors that affect OINV in cancer patients remain unclear. In this study, we developed a nomogram for predicting the occurrence of OINV in this population using retrospective clinical data.

**Methods:**

We collected data from 416 cancer pain patients, 70% of whom used the training set to analyze demographic and clinical variables. We used multivariate logistic regression to identify significant factors associated with OINV. Then, we construct a prediction nomogram. The validation set comprises the remaining 30%. The reliability of the nomogram is evaluated by bootstrap resampling.

**Results:**

Using multivariate logistic regression, we identified five significant factors associated with OINV. The C-index was 0.835 (95% confidence interval [CI], 0.828–0.842) for the training set and 0.810 (95% CI, 0.793–0.826) for the validation set. The calibrated curves show a good agreement between the predicted and actual occurrence of OINV.

**Conclusion:**

In a retrospective study based on five saliency-found variables, we developed and proved a reliable nomogram model to predict OINV in cancer pain patients. Future prospective studies should assess the model’s reliability and usefulness in clinical practice.

**Supplementary Information:**

The online version contains supplementary material available at 10.1007/s00520-023-08144-0.

## Introduction

Cancer is a prominent cause of mortality and a significant global obstacle to enhancing life expectancy [1]. The World Health Organization (WHO) estimated that in 2020, there were 19.3 million new cases and 10.0 million deaths [2]. Extraordinary advancements in diagnosis and treatment led to a substantial improvement in the five-year survival rate of cancer patients [3, 4]. Unfortunately, some painful experiences accompanying diagnosis and treatment can considerably impact the quality of life for cancer survivors [5]. Above all, pain is one of the most common symptoms of cancer and its treatment. Approximately 55% of individuals undergoing active treatment endure pain, while its prevalence exceeds 66% in those with advanced disease [6]. Cancer pain is highly agonizing, some patients endure prolonged suffering, often causing mental illness, and increasing the risk of suicidal tendencies. Poor management of cancer pain can significantly reduce the effectiveness of treatment and adversely affect the quality of life of patients [7]. The three-step analgesic ladder of the World Health Organization (WHO) has been widely extended in the treatment of cancer pain, resulting in adequate pain relief for the vast majority of patients [8]. Nevertheless, some individuals encounter challenges adhering to opioid therapy due to toxic side effects such as constipation, nausea, and vomiting [9, 10].

The prevalence of opioid-induced nausea and vomiting (OINV) is reported to be approximately 40% and 15–25%, respectively, although estimates may differ [11]. Severe nausea and vomiting not only inflict physical and psychological distress on patients but can also compromise their treatment adherence. Despite the high incidence rate and significant impact of OINV, there remains considerable controversy in current research regarding the use of prophylactic antiemetics versus treating nausea and vomiting after their occurrence [12–15]. The controversy may stem from a lack of predictive analysis regarding the risk of experiencing nausea and vomiting, as well as the absence of personalized assessments to determine the most effective treatment. Developing a personalized approach to managing opioid-induced nausea and vomiting (OINV) based on individual risk prediction is crucial. By identifying patients at a higher risk of developing OINV, it may be possible to guide the administration of antiemetics before initiating opioid therapy.

A multicenter study conducted in Europe reported that specific clinical characteristics and certain single nucleotide polymorphisms (SNPs) may contribute to the development of opioid-induced nausea and vomiting (OINV) [7]. Another study found associations between OINV and factors such as female sex, lung cancer, steroid use, age < 50 years, smoking, and gastrointestinal cancer [14]. There was also a cohort study that showed that age, edema, and gastrointestinal cancer were risk factors for OINV [16]. Although the above studies analyzed OINV-related risk factors, there is still a lack of mathematical models to anticipate and measure the probability of an event occurrence. As a result, the practical use of these findings is limited in a clinical setting.

Mathematical models have gained widespread use in predicting disease prognosis and treatment-related adverse reactions [17]. Out of all the models available, the nomogram model is the most effective visualization tool for regression equations. It establishes scoring criteria using regression coefficients of independent variables, which makes it easier to predict the probability of event occurrence [18]. A nomogram not only consolidates predictions from multiple variables but also accurately assesses the probability of a single event, resulting in its extensive application [19, 20]. This study aims to examine the risk factors associated with Opioid-induced nausea and vomiting (OINV) and develop a prediction model to identify patients who are susceptible to experiencing OINV when receiving opioid therapy. The objective is to facilitate the identification of high-risk populations for OINV through clinical screening and enable early antiemetic intervention. This personalized approach holds promise in reducing the occurrence and severity of OINV, thereby improving patient outcomes.

## Materials and methods

### Study design, population, and ethical considerations

The study was retrospective. Between June 2020 and June 2022, patients diagnosed with cancer pain who were prescribed opioids due to solid cancer by palliative care specialists were recruited from 3 different hospitals in Tianjin Medical University General Hospital, The First Hospital of Qinhuangdao, and People's Hospital of Inner Mongolia Autonomous Region. Data for 416 patients were available. All patients were randomly divided into the training group (*n* = 293) and internal validation group (*n* = 123) according to the proportion of 7:3.

The inclusion criteria were as follows: (1) patients who used either weak or strong opioids; (2) ≥ 18 years old with malignant solid tumor; (3) patients opioid naïve or not. the exclusion criteria were as follows: (1) the patient has serious emotional or mental disorders; (2) the patients have experienced nausea, retching, or vomiting in the 24 h prior to opioid use; (3) the patient had symptomatic primary or metastatic centers nervous system malignancy; (4) the patient had nausea and vomiting caused by intestinal obstruction, electrolyte disturbance, and metabolic abnormalities vomit; (5) the patient has a history of glaucoma and recent episodes are frequent; (6) a patient can be included in the study only once.

OINV diagnostic criteria are defined according to Common Adverse Event Terminology Assessment Standard 5.0 (CTCAE.v5.0). Nausea or vomiting greater than or equal to grade 1 in the CTCAE.v5.0 criteria are defined as the occurrence of opioid-related nausea or vomiting.

### Data collection

The general information of all cases was recorded in detail according to the electronic case data and the contents of the questionnaire collected Data, including gender (male, female), age, tumor type (gastrointestinal cancer, non-gastrointestinal cancer), history of drinking, history of motion sickness, average sleep time, anxiety level, thought to have side effects, first-time use of opioids (or not), drug dose adjustment, presence of CINV in this or previous chemotherapy. It should be noted that the treatment in CINV refers to conventional chemotherapy.

### Data quality control

In terms of data quality control, to ensure the scientific rigor and precision of the study, a stringent process will be followed to select research subjects based on inclusion and exclusion criteria. Prior to data collection, healthcare personnel will undergo professional training and adhere to a standardized set of guidelines to explain data entry requirements, thereby ensuring the quality of data collection. When encountering situations where it is unclear whether nausea and vomiting are caused by other factors, we will conduct a differential diagnosis of the factors causing nausea and vomiting, and it will only be included if the diagnosis is OINV. Unable to differentiate between opioid medications and other confounding factors causing nausea and vomiting will be excluded.

### Statistical analysis

The survey data was input and analyzed using SPSS v24.0 software (SPSS Inc., Chicago, IL, USA). Frequency and percentage were used to present the demographic data of patients and the incidence of OINV. Mann–Whitney *U* test was utilized to compare continuous data between the training group and the validation group, while the chi-square test was conducted to look for significant associations between category variables.

Firstly, in the training group, univariate logistic regression analysis was conducted on each variable to select variables with statistically significant differences. Subsequently, the variables with significant associations were incorporated into the multivariate logistic analysis to determine independent risk factors for OINV in patients. Finally, the selected independent risk factors were used to establish a nomogram for predicting the probability of OINV in patients. The nomogram translates each regression coefficient into a score ranging from 0 to 100. The variable with the lowest β coefficient is allocated a score of 100. The points associated with each independent variable are cumulatively summed to derive the overall point total, which is then transformed into predicted probabilities. (C-) index, also known as the Concordance Index, is a statistical measure commonly used to assess the predictive capability of a model. The (C-) index ranges from 0.5 (no discrimination, equivalent to random guessing) to 1.0 (perfect discrimination). The receiver operating characteristic (ROC) curve is a central metric used for evaluating the discriminative performance of predictive models [21]. It plots the true positive rate (sensitivity) against the false positive rate (1-specificity) at various classification thresholds. The area under the ROC curve (AUC) summarizes the overall performance of the model. AUC = 1 indicates perfect discrimination, 0.9 < AUC < 1 indicates excellent discrimination, 0.7 < AUC < 0.9 indicates a good discrimination, 0.5 < AUC < 0.7 indicates a poor discrimination, AUC = 0.5 is equivalent to random guessing, indicating no discrimination. In this study, the (C-)index and ROC curve were used to assess the nomogram’s predictive accuracy or discrimination. In order to enhance the clinical utility of the nomogram, optimal cut-off values for continuous variables were determined based on ROC curves and the Youden index [22]. A calibration plot was drawn to determine the consistency of the probabilities predicted by the nomogram and observation probabilities. Decision Curve Analysis (DCA) is a method used to evaluate the clinical utility or practical usefulness of a predictive model or diagnostic test [23]. It takes into account the benefits (true positives) and harms (false positives) of using a model across different threshold probabilities. So, the clinical benefits of the nomogram were estimated by the DCA.

Nomogram construction and validation were performed using the ‘rms’ and ‘car’ packages of R 4.1.1 software (https://cran.r-project.org/bin/windows/base/old/4.1.1/R-4.1.1-win.exe). In all statistical analyses, a level of *P* < 0.05 was adopted to determine statistical significance.

## Results

### Characteristics of the study population

A total of 416 subjects were collected and analyzed in this study (293 for nomogram development and 123 for validation), as shown in Table 1. The demographic and clinical characteristics of the study population are shown in Table 1. There was no statistical difference in most variables between the two data sets due to random allocation by R software. Only the *P* value of the History of motion sickness in the two groups was less than 0.05. Importantly, in the training set, OINV was reported in 17.7% of patients (52/293). In the validation set, 22% of patients (27/123) experienced OINV.

**Table 1:** Demographic and clinical characteristics of the training and validation sets

Variable	Total (416)	Training(293)	Validation(123)	Z/χ^2^	*P*
	*N*(%)	*N*(%)	*N*(%)		
Gender					
Male	276 (66.3)	197 (67.2)	79 (64.2)	0.351	0.554
Female	140 (33.7)	96 (32.8)	44 (35.8)		
Age, years					
	65(58.25–70)	65(58–70)	64(59–69)	–0.745	0.456
> 60	291 (70.0)	203 (69.3)	88 (71.5%)	0.211	0.646
≤ 60	125 (30.0)	90 (30.7)	35 (28.5)		
Tumor type					
Gastrointestinal cancer	104 (25.0)	72 (24.6)	32 (26.0)	0.096	0.756
Non-gastrointestinal cancer	312(75.0)	221 (75.4)	91 (74.0)		
History of drinking					
Yes	165(39.7)	121 (41.3)	44 (35.8)	1.105	0.293
No	251(60.3)	172 (58.7)	79 (64.2)		
History of morning sickness					
Yes	68(48.6)	49 (51.0)	19 (43.2)	0.746	0.388
No	72(51.4)	47 (49.0)	25 (56.8)		
History of motion sickness					
Yes	72(17.3)	58 (19.8)	14 (11.4)	4.284	0.038^*^
No	344(82.7)	235 (80.2)	109 (88.6)		
Anxiety					
No	183(44.0)	128 (43.7)	55 (44.7)	2.439	0.486
Mild	186(44.7)	134 (45.7)	52 (42.3)		
Moderate	36(8.7)	22 (7.5)	14 (11.4)		
Severe	11(2.6)	9 (3.1)	2 (1.6)		
Average sleep time					
< 5 h	129(31.0)	91 (31.1)	38 (30.9)	0.536	0.911
5–7 h	228(54.8)	161 (54.9)	67 (54.5)		
8–9 h	46(11.1)	33 (11.3)	13 (10.6)		
> 9 h	13(3.1)	8 (2.7)	5 (4.1)		
Thought to have side effects					
Yes	100(24.0)	64 (21.8)	36 (29.3)	2.726	0.256
No	110(26.4)	81 (27.6)	29 (23.6)		
Unclear	206(49.5)	148 (50.5)	58 (47.2)		
First-time use of opioids					
Yes	209(50.2)	144 (49.1)	65 (52.8)	0.474	0.491
No	207(49.8)	149 (50.9)	58 (47.2)		
Drug dose adjustment					
Yes	164(39.4)	115 (39.2)	49 (39.8)	0.013	0.911
No	252(60.6)	178 (60.8)	74 (60.2)		
Presence of CINV in this or previous chemotherapy					
Yes	65(15.6)	41 (14.0)	24(19.5)	2.002	0.157
No	351(84.4)	252 (86.0)	99(80.5)		
Type of opioid					
Morphine sustained-release tablet	47(11.3)	32(10.9)	15(12.2)		
Oxycodone extended-release tablet	290(69.7)	206(70.3)	84(68.3)	1.174	0.759
Fentanyl transdermal patches	33(7.9)	21(7.2)	12(9.8)		
Others	46(11.1)	34(11.6)	12(9.8)		
Incidence of OINV					
Yes	79(19.0)	52(17.7)	27(22)	0.995	0.318
No	337(81.0)	241(82.3)	96(78)		

### Risk factors for OINV of patients

We first determine the predictive value of each variable through logistic regression analysis. Table 2 describes the univariate logistic regression analysis of OINV. Unifactorial analysis gender (female), age (≤ 60 years old), history of motion sickness, average sleep time < 5 h at night, non-first-time use of opioids, presence of CINV in this or previous chemotherapy, drug dose adjustment and thought to have side effects were significantly associated with OINV. However, no statistically significant relationships (*P* > 0.05) were observed with a history of drinking, history of morning sickness (only female), anxiety, gastrointestinal cancer, or type of opioid.

**Table 2:** Univariate logistic regression analysis of OINV in patients

Variable	OR	95%CI	*P* value
Gender			
Male	1 (Reference)		
Female	2.012	1.092–3.707	0.025
Age, years			
≤ 60	1 (Reference)		
> 60	0.968	0.94–0.997	0.029
History of drinking			
Yes	1 (Reference)		
No	1.154	0.624–2.134	0.124
History of morning sickness			
Yes	1 (Reference)		
No	0.523	0.203–1.347	0.179
History of motion sickness			
Yes	1 (Reference)		
No	0.239	0.124–0.461	< 0.0001
Average sleep time			
< 5 h	1(Reference)		
5–7 h	0.44	0.233–0.833	0.012
8–9 h	0.264	0.074–0.943	0.040
> 9 h	0.377	0.044–3.222	0.373
Anxiety			
No	1 (Reference)		
Mild	1.426	0.757–2.687	0.272
Moderate	0.853	0.231–3.153	0.811
Severe	0.675	0.080–5.697	0.718
Thought to have side effects			
Yes	1 (Reference)		
No	0.334	0.142–0.782	0.012
Unclear	0.436	0.217–0.875	0.019
First-time use of opioids			
Yes	1 (Reference)		
No	3.573	1.816–7.031	< 0.0001
Drug dose adjustment			
Yes	1 (Reference)		
No	0.328	0.177–0.609	< 0.0001
Presence of CINV in this or previous chemotherapy			
Yes	1 (Reference)		
No	0.117	0.057–0.24	< 0.0001
Tumor type			
Gastrointestinal cancer	1 (Reference)		
Non-gastrointestinal cancer	0.545	0.286–1.041	0.066
Type of opioid			
Morphine sustained-release tablet	1 (Reference)		
Oxycodone extended-release tablet	0.797	0.304–2.091	0.645
Fentanyl transdermal patches	1.733	0.473–6.346	0.406
Others	1.333	0.406–4.382	0.636

Based on the analysis results, we conducted a multivariate logistic regression analysis, as shown in Table 3. History of motion sickness, average sleep time < 5 h at night, non-first-time use of opioids, presence of CINV in this or previous chemotherapy, and drug dose adjustment were significantly associated with OINV, which were used in the construction of the nomogram. Notably, age, gender, and gastrointestinal cancer which were important predictors of OINV in cancer pain patients [7, 16], showed no significant association with the occurrence of OINV in the study.

**Table 3:** Variables significantly associated with OINV from the multivariate logistic regression model

Variable	OR	95%CI	*P* value
History of motion sickness			
Yes	1 (Reference)		
No^*^	0.345	0.155–0.765	0.009
Average sleep time			
< 5 h	1(Reference)		
≥ 5 h and < 7 h^*^	0.380	0.176–0.817	0.013
≥ 7 h^*^	0.321	0.084–1.229	0.097
First-time use of opioids			
Yes	1 (Reference)		
No^#^	4.117	1.850–9.165	0.001
Drug dose adjustment			
Yes	1 (Reference)		
No^*^	0.359	0.171–0.755	0.007
Presence of CINV in this or previous chemotherapy			
Yes	1 (Reference)		
No^*^	0.118	0.051–0.276	< 0.0001

^*^ Represents a decreased risk

^#^Represents an increase in risk

In order to identify whether there are any other significant risk factors, we conducted both univariate and multivariate logistic regression analyses separately for nausea and vomiting as outcomes in the overall population (Supplementary Table S1–4). Compared to these, the independent risk factors for OINV are more comprehensive.

### OINV nomogram construction

**Fig. 1:**
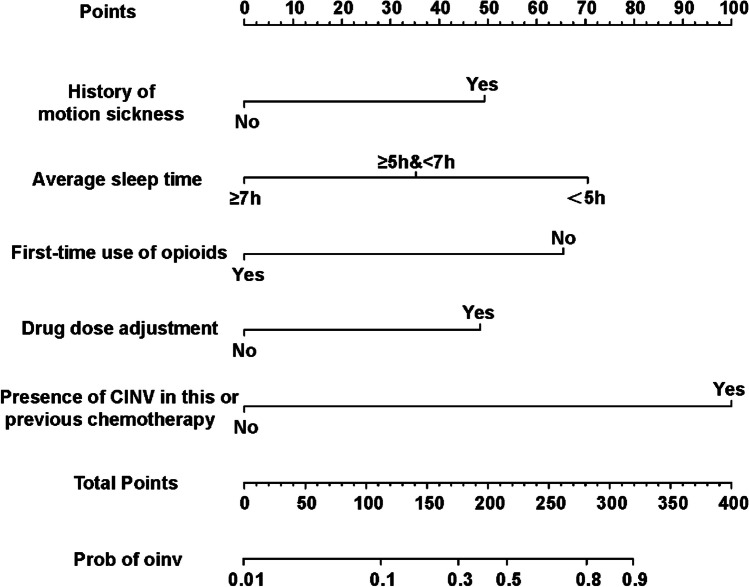
Prediction nomogram of OINV in patients receiving opioid treatment. The nomogram is used by summing all points identified on the scale for each variable. The total points projected on the bottom scales indicate the probabilities of OINV

**Fig. 2:**
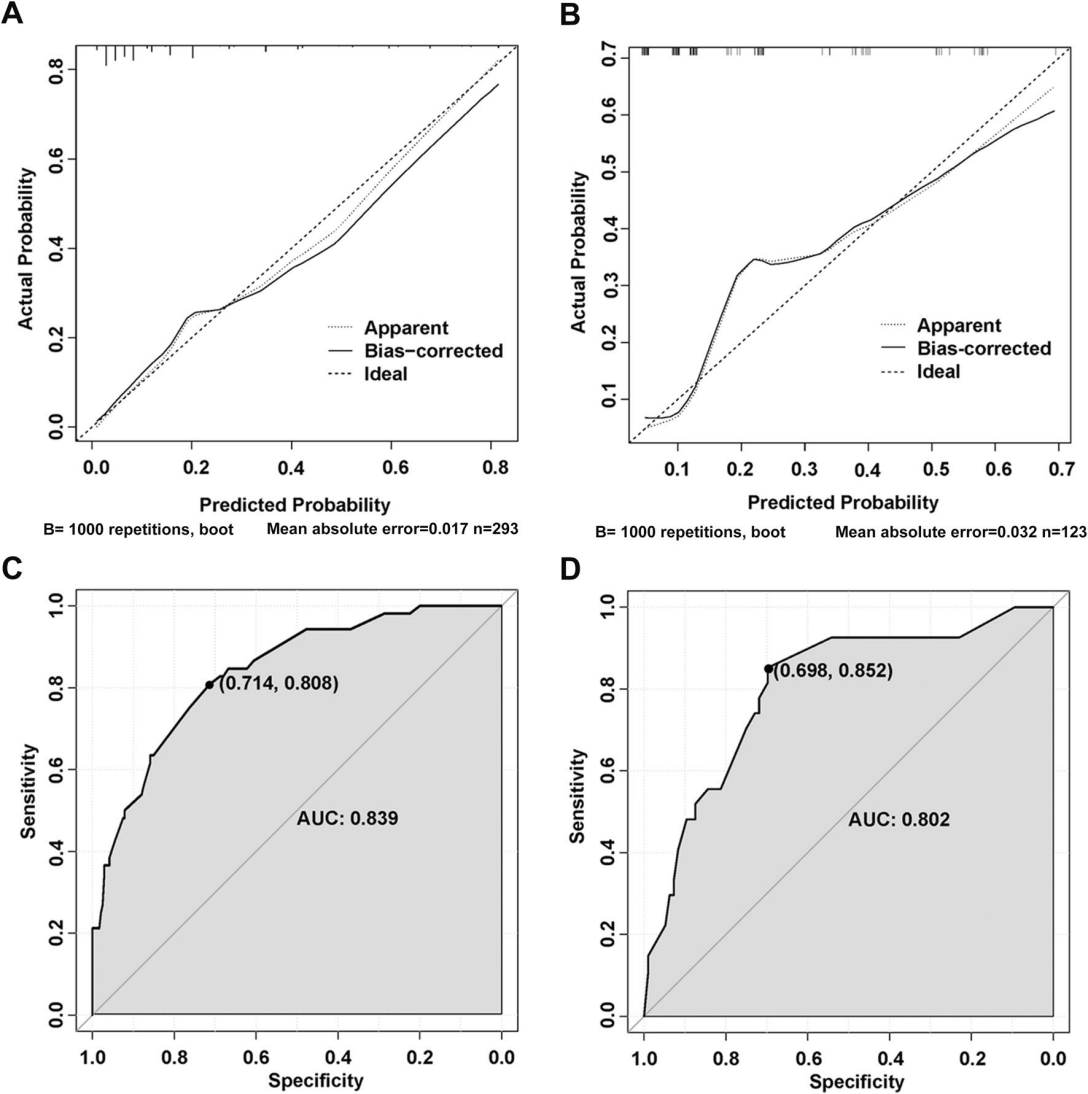
Calibration plot and ROC curve of the nomogram. **A** Training set. **B** Validation set. The *X*-axis represents the predicted OINV probabilities estimated using the nomogram, while the *Y*-axis displays the actual rates of OINV. The solid straight line serves as an ideal reference line, indicating a perfect correspondence between predicted OINV and actual outcome. Additionally, the dashed straight lines represent a 10% margin of error. **C**, **D** ROC curve for discrimination in the training and validation cohorts; the AUC was 0.839 (95% CI, 0.780–0.897) and 0.802 (95% CI, 0.708–0.897), respectively, indicating that the model has good predictive value

We established a nomogram based on the logistic regression model developed using data from the training set. Significant risk factors identified by logistic regression analysis were fitted into the model. The final nomogram is shown in Fig. 1. Validation was first performed with the training set. Bootstrapping (1000 replications) was applied and a calibration curve was generated (Fig. 2A). There was no obvious deviation between the risk curve predicted by the model and the actual observed risk, indicating that the model is well-calibrated. The C-index for OINV prediction was 0.839 (95% CI, 0.832–0.845) in this nomogram, the area under the ROC curve (AUC) is 0.839 (95% CI, 0.780–0.897) (Fig. 2C), indicating a good performance of OINV prediction using the combined nomograms. Calculating the Youden index using the ROC curve to define the cut-off threshold was 136, corresponding to 80.8% sensitivity, and 71.4% specificity. When the total score is below 136, patients are less likely to be diagnosed with OINV.

### Validation of the OINV nomogram

The validation set data of patients (*n* = 123) were used for model validation. The calibration plot for the probability of OINV revealed good concordance between the nomogram prediction and actual observations (Fig. 2B). The C-index for OINV prediction was 0.802 (95% CI, 0.786–0.819) and the AUC was 0.802 (95% CI, 0.708–0.897), demonstrating that the nomogram was well-fitted (Fig. 2D).

### DCA for clinical utility of the established nomograms

**Fig. 3:**
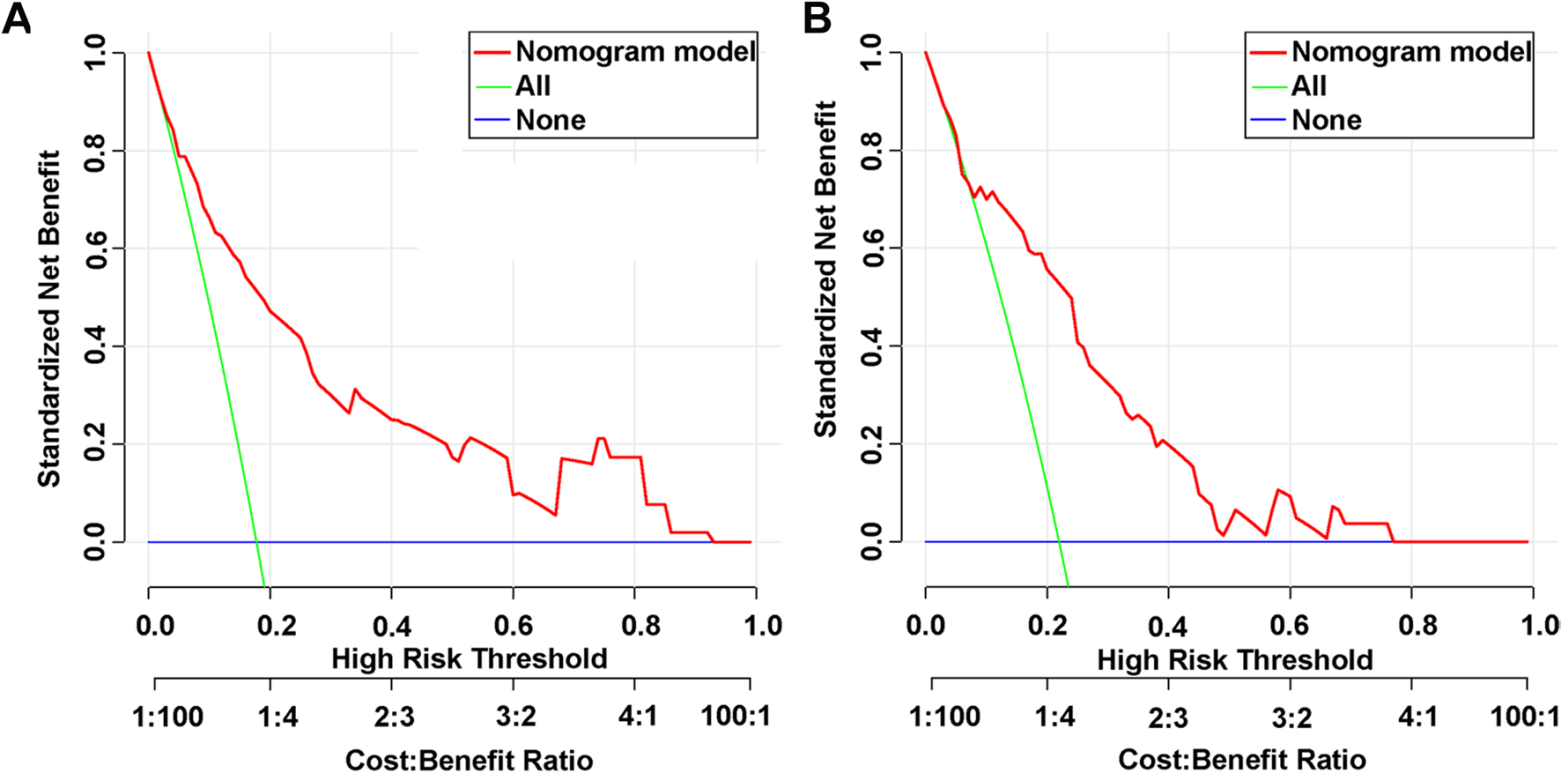
Decision curve analysis (DCA) of the nomogram model for predicting OINV. **A** training set, **B** validation set. The abscissa shows the threshold probability of the OINV prediction, and the ordinate indicates the net benefit of the benefits and the hazards. The black parallel horizontal line above the abscissa indicates that none of the patients died and the net benefit is 0. The grey line indicates that OINV occurred in all patients

The decision curve analysis (DCA) was subsequently performed to illustrate the clinical decision utility of the combined nomogram. As shown in Fig. 3A, the threshold probabilities for the train set range from 5 to 92%, and the screening strategy based on our nomogram OINV risk estimate results in a superior net benefit compared to the treat-none or treat-all strategies. In the validation set, the threshold probability is between 15 and 78% (Fig. 3B). Consequently, DCA reveals that the nomogram has a superior overall net benefit over a wide range of practical threshold probabilities, indicating a high potential clinical utility.​

## Discussion

As there are no established criteria for diagnosing OINV, patients must report their subjective experience of nausea. Therefore, it is essential to consider the characteristics of the symptoms, including their frequency, intensity, and duration, as well as their quantitative and qualitative aspects. However, consensus on the ideal assessment tool and the optimal assessment period is yet to be reached, resulting in variations in the reported incidence of OINV across numerous clinical studies, ranging from 4 to 60% [8, 15, 24, 25]. In this study, we used the Common Adverse Event Terminology Assessment Standard version 5.0 (CTCAE.v5.0) to define OINV. The analysis showed that the incidence of OINV in 416 cases in this study was 18.9%. This is consistent with previous reports [16, 26]. Therefore, our findings may be implemented in general scenarios.

In our study, we found no significant correlation between age and gender, and OINV. However, previous studies [9, 14] have suggested that female patients are more susceptible to OINV than male patients. This finding is inconsistent with our study. However, a retrospective study [16] found that there was no statistical difference in the incidence of OINV in terms of gender, so the mechanism of promoting the incidence of OINV by female patients still needs to be further explored. A higher frequency of OINV had been reported in patients < 50 years. However, a retrospective study of postoperative nausea and vomiting caused by opioids found that there was no significant difference in age between patients using opioids and control [27]. Our study also showed that there was no significant difference between the occurrence of OINV and age. However, it should not be ignored that in this study, the median age (inter-quartile range) of the overall patients was 65(58.25–70), mainly elderly patients, which is also one of the factors affecting the analysis results.

The multivariate logistic regression analysis used in this study demonstrated that history of motion sickness, mean sleep duration < 5 h at night, non-first-time use of opioids, presence of CINV in this or previous chemotherapy, and drug dose adjustment were closely associated with the occurrence of OINV. At present, there are no reports of using motion sickness and CINV history to predict OINV occurrence. However, the occurrence of nausea and vomiting is often caused by conditional stimulation, patients who have had nausea and vomiting in the past are more likely to have nausea and vomiting again when they are subjected to the same stimulation [28]. This could also explain why patients with motion sickness and a history of CINV in our study were led to an increased risk of OINV. It was also confirmed that poor sleep quality was significantly associated with nausea and vomiting [29]. Our findings align with this conclusion. Interestingly, our study implied that patients with non-first-time use of opioids and drug dose adjustment are at higher risk of experiencing OINV, which has not been reported in previous studies. Understandably, increasing the dosage to alleviate pain can lead to a higher likelihood of experiencing OINV. However, the mechanism of first-time use of opioids as a protective factor for OINV still needs further investigation.

To our knowledge, there have been no reports on the development and validation of a model that predicts the risk of OINV in cancer patients. This study has created a nomogram for predicting the occurrence of OINV in patients with cancer pain, based on five independent risk factors identified through binary logistic regression analysis. By calculating the total score of the individual scores of each risk factor, the nomogram can accurately predict the risk of OINV in these patients. According to the C-index, the prediction model for OINV risk had an accuracy of 0.835 in the training set. Our forecasting model has a higher level of accuracy, as the CINV forecasting model is only 0.78 or lower [30, 31]. Unfortunately, there is no OINV forecasting model available for comparison. In the validation set, the C-index was 0.810, supporting the validity of the predictive model. To better evaluate the practical application of the prediction model, the ROC curve is used in this study as it provides a more intuitive analysis. The differentiation degree of the prediction model is evaluated using this curve. Typically, an AUC value greater than 0.7 is considered to indicate good discriminatory power. The results show that the AUC of the prediction model in this study is 0.835. The 95% confidence interval is (0.828–0.842), so this model can distinguish well whether OINV occurs.

Moreover, the Bootstrap self-extraction method was utilized in this study for internal validation of the prediction model about high-risk factors of OINV. The correction curve exhibited a diagonal relationship, indicating strong concordance between the predicted probability of OINV occurrence in this study model and the actual probability. Overall, the model demonstrates enhanced capability in identifying high-risk patients susceptible to OINV. In clinical practice, healthcare professionals can utilize the risk prediction model to promptly identify cancer pain patients with a high likelihood of experiencing OINV. This enables timely preventive antiemetic interventions and personalized measures to alleviate patient discomfort. Hence, the prediction model we have developed has the potential to serve as a robust foundation for clinical interventions in patients, while also providing a sound theoretical framework for guideline revision.

## Study limitation

The aforementioned method exhibits certain limitations and necessitates further refinement before its implementation in clinical practice. The study design was retrospective and focused on a multicenter sample, but the majority of the sample size was from a single center, necessitating further validation through a larger multicenter sample and the inclusion of an external validation set. In addition, this study included only Chinese subjects. Although there have been reports of differences in opioid receptors among Asian patients, no studies have yet investigated racial disparities in OINV [32]. Before widely applying the findings of this study, it may be necessary to validate any potential racial disparities. Finally, in the course of electronic questionnaire data collection, certain patients recorded their pain scores subsequent to the ingestion of oral opioids. Consequently, this specific dataset subset was not incorporated into the dataset for comprehensive analysis. In future research endeavors, we intend to optimize our data collection protocols to encompass the pain scores within the analytical domain.

## Conclusion

We developed a nomogram model to predict OINV in cancer pain patients. The nomogram has good prediction performance in both training and validation cohorts for estimating the risk of OINV. Future prospective studies should assess the model’s reliability and usefulness in clinical practice.

### Supplementary Information

Below is the link to the electronic supplementary material.

Supplementary file1 (DOCX 27 KB)

## Data Availability

The data used to support the findings of this study are available from the corresponding author upon request.

## References

[1] Bray F, Laversanne M, Weiderpass E, Soerjomataram I (2021). The ever-increasing importance of cancer as a leading cause of premature death worldwide. Cancer..

[2] Sung H, Ferlay J, Siegel RL, Laversanne M, Soerjomataram I, Jemal A, Bray F (2021). Global Cancer Statistics 2020: GLOBOCAN Estimates of Incidence and Mortality Worldwide for 36 Cancers in 185 Countries. CA Cancer J Clin..

[3] Zhu C, Ma H, He A, Li Y, He C, Xia Y (2022). Exercise in cancer prevention and anticancer therapy: Efficacy, molecular mechanisms and clinical information. Cancer Lett..

[4] Bharti R, Dey G, Lin F, Lathia J, Reizes O (2022). CD55 in cancer: Complementing functions in a non-canonical manner. Cancer Lett..

[5] Yu S, Li W, Tang L, Fan X, Yao S, Zhang X, Bi Z, Cheng H (2022). Depression in breast cancer patients: Immunopathogenesis and immunotherapy. Cancer Lett..

[6] van den Beuken-van Everdingen MH, Hochstenbach LM, Joosten EA, Tjan-Heijnen VC, Janssen DJ (2016). Update on prevalence of pain in patients with cancer: systematic review and meta-analysis. J Pain Symptom Manage..

[7] Laugsand EA, Fladvad T, Skorpen F, Maltoni M, Kaasa S, Fayers P, Klepstad P (2011). Clinical and genetic factors associated with nausea and vomiting in cancer patients receiving opioids. Eur J Cancer.

[8] Sande TA, Laird BJA, Fallon MT (2019). The management of opioid-induced nausea and vomiting in patients with cancer: a systematic review. J Palliat Med.

[9] Cepeda MS, Farrar JT, Baumgarten M, Boston R, Carr DB, Strom BL (2003). Side effects of opioids during short-term administration: effect of age, gender, and race. Clin Pharmacol Ther.

[10] Quigley C (2005). The role of opioids in cancer pain. BMJ.

[11] Coluzzi F, Rocco A, Mandatori I, Mattia C (2012). Non-analgesic effects of opioids: opioid-induced nausea and vomiting: mechanisms and strategies for their limitation. Curr Pharm Des..

[12] Laugsand EA, Kaasa S, Klepstad P (2011). Management of opioid-induced nausea and vomiting in cancer patients: systematic review and evidence-based recommendations. Palliat Med.

[13] McNicol E, Horowicz-Mehler N, Fisk RA, Bennett K, Gialeli-Goudas M, Chew PW, Lau J, Carr D, Americal Pain S (2003). Management of opioid side effects in cancer-related and chronic noncancer pain: a systematic review. J Pain..

[14] Kanbayashi Y, Hosokawa T (2014). Predictive factors for nausea or vomiting in patients with cancer who receive oral oxycodone for the first time: is prophylactic medication for prevention of opioid-induced nausea or vomiting necessary?. J Palliat Med..

[15] Sato J, Tanaka R, Ishikawa H, Suzuki T, Shino M (2020). A preliminary study of the effect of naldemedine tosylate on opioid-induced nausea and vomiting. Support Care Cancer..

[16] Ogawa Y, Kurihara T, Sakurai M, Monma M, Nakayama H, Higuchi H, Kogo M, Kiuchi Y (2021). Predictive factors of opioid-induced nausea in cancer patients. J Pain Palliat Care Pharmacother..

[17] Zhang Q, Ouyang H, Ye F, Chen S, Xie L, Zhao X, Yu X (2020). Multiple mathematical models of diffusion-weighted imaging for endometrial cancer characterization: correlation with prognosis-related risk factors. Eur J Radiol..

[18] Kattan MW, Scardino PT (2007). Evidence for the usefulness of nomograms. Nat Clin Pract Urol..

[19] Kattan MW (2002). Nomograms. Introduction Semin Urol Oncol.

[20] Li Q, Jiang T, Zhang C, Zhang Y, Huang Z, Zhou H, Huang P (2022). A nomogram based on clinical information, conventional ultrasound and radiomics improves prediction of malignant parotid gland lesions. Cancer Lett.

[21] Alba AC, Agoritsas T, Walsh M, Hanna S, Iorio A, Devereaux PJ, McGinn T, Guyatt G (2017). Discrimination and calibration of clinical prediction models: users' guides to the medical literature. JAMA..

[22] Zheng X, He X (2023). Development of a nomogram for the prediction of complicated appendicitis during pregnancy. BMC Surg..

[23] Vickers AJ, Elkin EB (2006). Decision curve analysis: a novel method for evaluating prediction models. Med Decis Making..

[24] Corli O, Santucci C, Corsi N, Radrezza S, Galli F, Bosetti C (2019). The burden of opioid adverse events and the influence on cancer patients' symptomatology. J Pain Symptom Manage..

[25] Tsukuura H, Miyazaki M, Morita T, Sugishita M, Kato H, Murasaki Y, Gyawali B, Kubo Y, Ando M, Kondo M, Yamada K, Hasegawa Y, Ando Y (2018). Efficacy of Prophylactic Treatment for Oxycodone-Induced Nausea and Vomiting Among Patients with Cancer Pain (POINT): A Randomized, Placebo-Controlled Double-Blind Trial. Oncologist.

[26] Walsh D, Davis M, Ripamonti C, Bruera E, Davies A, Molassiotis A (2017). 2016 Updated MASCC/ESMO consensus recommendations: Management of nausea and vomiting in advanced cancer. Support Care Cancer..

[27] Ukai T, Ebihara G, Watanabe M (2018). Opioid administration via epidural catheter is a risk factor for postoperative nausea and vomiting in total hip arthroplasty: A retrospective study. J Orthop Sci..

[28] Molassiotis A, Stamataki Z, Kontopantelis E (2013). Development and preliminary validation of a risk prediction model for chemotherapy-related nausea and vomiting. Support Care Cancer..

[29] Jung D, Lee KM, Kim WH, Lee JY, Kim TY, Im SA, Lee KH, Spiegel D, Hahm BJ (2016). Longitudinal association of poor sleep quality with chemotherapy-induced nausea and vomiting in patients with breast cancer. Psychosom Med..

[30] Huang XJ, Li XY, Li JH, Hu ZY, Luo L, Tan Y, Chen HY, Fan RR, Wang TY, Meng LQ, Wei T (2021). Nomogram for predicting chemotherapy-induced nausea and vomiting for breast cancer patients. Tohoku J Exp Med..

[31] Dranitsaris G, Molassiotis A, Clemons M, Roeland E, Schwartzberg L, Dielenseger P, Jordan K, Young A, Aapro M (2017). The development of a prediction tool to identify cancer patients at high risk for chemotherapy-induced nausea and vomiting. Ann Oncol..

[32] Bond C, LaForge KS, Tian M, Melia D, Zhang S, Borg L, Gong J, Schluger J, Strong JA, Leal SM, Tischfield JA, Kreek MJ, Yu L (1998). Single-nucleotide polymorphism in the human mu opioid receptor gene alters beta-endorphin binding and activity: possible implications for opiate addiction. Proc Natl Acad Sci U S A..

