# Supplementary material for: Nomogram for predicting opioid-induced nausea and vomiting for cancer pain patients
Source: Support Care Cancer. 2023 Nov 2;31(11):663. doi: 10.1007/s00520-023-08144-0 (PMC10620250; doi:10.1007/s00520-023-08144-0)
Supplement: Supplementary file 1 — Supplementary file1 (DOCX 27 KB) [file 520_2023_8144_MOESM1_ESM.docx]

The independent risk factors for the nausea group are as follows: History of motion sickness, Average sleep time, First-time use of opioids, Drug dose adjustment (Sup. Table 1,3). And the independent risk factors for the vomiting group are as follows: History of morning sickness, Drug dose adjustment, First-time use of opioids (Sup. Table 2,4).

Table 1 Univariate logistic regression analysis of nausea in patients (n=416)

| Variable | OR | 95%CI | *P* value |
| --- | --- | --- | --- |
| Gender |  |  |  |
| Male | 1 (Reference) |  |  |
| Female | 1.768 | 1.059-2.950 | 0.029 |
| Age, years |  |  |  |
| ≤60 | 1 (Reference) |  |  |
| >60 | 0.526 | 0.313-0.884 | 0.015 |
| History of drinking |  |  |  |
| Yes | 1 (Reference) |  |  |
| No | 1.358 | 0.801-2.303 | 0.255 |
| History of morning sickness |  |  |  |
| Yes | 1 (Reference) |  |  |
| No | 0.623 | 0.283-1.369 | 0.239 |
| History of motion sickness |  |  |  |
| Yes | 1 (Reference) |  |  |
| No | 0.264 | 0.149-0.456 | <0.0001 |
| Average sleep time |  |  |  |
| ＜5h | 1（Reference） |  |  |
| 5-7h | 0.471 | 0.276-0.802 | 0.006 |
| 0.0068-9h | 0.256 | 0.085-0.766 | 0.015 |
| ＞9h | 0.224 | 0.028-1.785 | 0.158 |
| Anxiety |  |  |  |
| No | 1 (Reference) |  |  |
| Mild | 1.677 | 0.967-2.906 | 0.066 |
| Moderate | 1.806 | 0.740-4.405 | 0.194 |
| Severe | 1.404 | 0.287-6.881 | 0.675 |
| Thought to have side effects |  |  |  |
| Yes | 1 (Reference) |  |  |
| No | 0.247 | 0.116-0.525 | <0.0001 |
| Unclear | 0.409 | 0.232-0.722 | 0.002 |
| First-time use of opioids |  |  |  |
| Yes | 1 (Reference) |  |  |
| No | 2.852 | 1.658-4.903 | <0.0001 |
| Drug dose adjustment |  |  |  |
| Yes | 1 (Reference) |  |  |
| No | 0.321 | 0.191-0.539 | <0.0001 |
| Presence of CINV in this or previous chemotherapy |  |  |  |
| Yes | 1 (Reference) |  |  |
| No | 1.591 | 1.089-2.323 | 0.016 |
| Tumor type |  |  |  |
| gastrointestinal cancer | 1 (Reference) |  |  |
| non-gastrointestinal cancer | 0.589 | 0.342-1.014 | 0.056 |
| Type of opioid |  |  |  |
| Morphine sustained-release tablet | 1 (Reference) |  |  |
| Oxycodone extended-release tablet | 0.617 | 0.293-1.300 | 0.204 |
| Fentanyl Transdermal Patches | 0.881 | 0.301-2.578 | 0.817 |
| Others | 0.909 | 0.344-2.405 | 0.848 |

Table 2 Univariate logistic regression analysis of vomiting in patients (n=416)

| Variable | OR | 95%CI | *P* value |
| --- | --- | --- | --- |
| Gender |  |  |  |
| Male | 1 (Reference) |  |  |
| Female | 2.398 | 1.182-4.862 | 0.015 |
| Age, years |  |  |  |
| ≤60 | 1 (Reference) |  |  |
| >60 | 0.449 | 0.221-0.913 | 0.027 |
| History of drinking |  |  |  |
| Yes | 1 (Reference) |  |  |
| No | 1.639 | 0.762-3.523 | 0.206 |
| History of morning sickness |  |  |  |
| Yes | 1 (Reference) |  |  |
| No | 0.316 | 0.106-0.940 | 0.038 |
| History of motion sickness |  |  |  |
| Yes | 1 (Reference) |  |  |
| No | 0.295 | 0.140-0.622 | 0.001 |
| Average sleep time |  |  |  |
| ＜5h | 1（Reference） |  |  |
| 5-7h | 0.574 | 0.274-1.203 | 0.141 |
| 8-9h | 0.345 | 0.076-1.573 | 0.169 |
| ＞9h | 0.633 | 0.077-5.223 | 0.671 |
| Anxiety |  |  |  |
| No | 1 (Reference) |  |  |
| Mild | 1.740 | 0.775-3.909 | 0.180 |
| Moderate | 2.790 | 0.893-8.721 | 0.078 |
| Severe | 3.844 | 0.731-20.209 | 0.112 |
| Thought to have side effects |  |  |  |
| Yes | 1 (Reference) |  |  |
| No | 0.184 | 0.060-0.568 | 0.003 |
| Unclear | 0.329 | 0.153-0.708 | 0.004 |
| First-time use of opioids |  |  |  |
| Yes | 1 (Reference) |  |  |
| No | 4.329 | 1.840-10.180 | 0.001 |
| Drug dose adjustment |  |  |  |
| Yes | 1 (Reference) |  |  |
| No | 0.206 | 0.093-0.454 | <0.0001 |
| Presence of CINV in this or previous chemotherapy |  |  |  |
| Yes | 1 (Reference) |  |  |
| No | 1.245 | 0.718-2.157 | 0.435 |
| Tumor type |  |  |  |
| gastrointestinal cancer | 1 (Reference) |  |  |
| non-gastrointestinal cancer | 0.582 | 0.277-1.221 | 0.152 |
| Type of opioid |  |  |  |
| Morphine sustained-release tablet | 1 (Reference) |  |  |
| Oxycodone extended-release tablet | 0.754 | 0.245-2.322 | 0.622 |
| Fentanyl Transdermal Patches | 2.389 | 0.617-9.248 | 0.207 |
| Others | 1.311 | 0.329-5.225 | 0.701 |

Table 3 Variables significantly associated with nausea from the multivariate logistic regression model (n=416)

| Variable | OR | 95%CI | *P* value |
| --- | --- | --- | --- |
| History of motion sickness |  |  |  |
| Yes | 1 (Reference) |  |  |
| No | 0.293 | 0.158-0.543 | <0.0001 |
| Average sleep time |  |  |  |
| <5h | 1（Reference） |  |  |
| ≥5h | 0.550 | 0.356-0.851 | 0.007 |
| First-time use of opioids |  |  |  |
| Yes | 1 (Reference) |  |  |
| No | 2.963 | 1.656-5.302 | <0.0001 |
| Drug dose adjustment |  |  |  |
| Yes | 1 (Reference) |  |  |
| No | 0.410 | 0.236-0.712 | 0.002 |

Table 4 Variables significantly associated with vomiting from the multivariate logistic regression model (n=416)

| Variable | OR | 95%CI | *P* value |
| --- | --- | --- | --- |
| History of morning sickness |  |  |  |
| Yes | 1 (Reference) |  |  |
| No | 0.284 | 0.090-0.898 | 0.032 |
| First-time use of opioids |  |  |  |
| Yes | 1 (Reference) |  |  |
| No | 4.616 | 1.206-17.677 | 0.026 |
| Drug dose adjustment |  |  |  |
| Yes | 1 (Reference) |  |  |
| No | 0.257 | 0.081-0.808 | 0.020 |
